# Supplementary material for: The genome sequence of the click beetle, Hemicrepidius hirtus (Herbst, 1784) (Coleoptera: Elateridae)
Source: Wellcome Open Res. 2025 Oct 3;10:536. [Version 1] doi: 10.12688/wellcomeopenres.24924.1 (PMC12759277; doi:10.12688/wellcomeopenres.24924.1)
Supplement: Peer review history

# Reviewer report: Reviewer response for version 1

**DOI:** 10.21956/wellcomeopenres.27454.r141507

**Published:** 2026-01-05

**Authors:** Chenyang Cai

**Affiliations:** 1 Nanjing Institute of Geology and Palaeontology Chinese Academy of Sciences, Nanjing, Jiangsu, China

This manuscript presents a high-quality, chromosome-level genome assembly for *Hemicrepidius hirtus*, the only UK species of its genus and an ecologically noteworthy click beetle. The study follows the established Darwin Tree of Life (DToL) workflows. Please clarify whether the mitochondrial genome was validated using coverage depth or annotation completeness.

 For antennal characters: *1st antennal segment 2nd segment shorter than the 4th segment, but the 2nd and 3rd antennal segments together as long as the 4th segment;*

*‘segment’ should be replaced by ‘antennomere’*

Are sufficient details of methods and materials provided to allow replication by others?

Yes

Is the rationale for creating the dataset(s) clearly described?

Yes

Are the datasets clearly presented in a useable and accessible format?

Yes

Are the protocols appropriate and is the work technically sound?

Yes

Reviewer Expertise:

phylogenomics

I confirm that I have read this submission and believe that I have an appropriate level of expertise to confirm that it is of an acceptable scientific standard.

# Reviewer report: Reviewer response for version 1

**DOI:** 10.21956/wellcomeopenres.27454.r141505

**Published:** 2026-01-02

**Authors:** Zachary Cohen

**Affiliations:** 1 Florida Atlantic University College of Engineering and Computer Science (Ringgold ID: 306684), Boca Raton, Florida, USA

Another impressive report on the industry standard for genome assemblies. By providing the genome for the only extant species in the Hemicrepidius genus, these data will be invaluable for further understanding on species diversity & the consequence of isolation. This work expertly leverages HiFi and Hi-C reads to generate a chromosome level genome assembly while isolating the mitochondria and X chromosome. Additionally, they provide the community with RNA seq data for subsequent analysis.

Are sufficient details of methods and materials provided to allow replication by others?

Yes

Is the rationale for creating the dataset(s) clearly described?

Yes

Are the datasets clearly presented in a useable and accessible format?

Yes

Are the protocols appropriate and is the work technically sound?

Yes

Reviewer Expertise:

genomics, entomology, adapation

I confirm that I have read this submission and believe that I have an appropriate level of expertise to confirm that it is of an acceptable scientific standard.

# Reviewer report: Reviewer response for version 1

**DOI:** 10.21956/wellcomeopenres.27454.r141510

**Published:** 2025-12-30

**Authors:** João Pedro Marques, Juliana Sofia Alves

**Affiliations:** 1 BIOPOLIS Association - Research Network on Biodiversity and Evolution Biology, Vairão, Porto, Portugal; 2 University of Porto Faculty of Sciences, Porto, Porto District, Portugal

The manuscript presents a high-quality chromosome-level genome assembly for *Hemicrepidius hirtus*. The genome was generated using current state-of-the-art methods within the Darwin Tree of Life project and is accompanied by a clear and thorough technical description.

 Overall, the manuscript is well written, technically sound, and fully appropriate for a genome note. The assembly quality is very high and meets recommended community standards. This genome will be a valuable resource for future studies on beetle genomics, evolution, and ecology. Our comments below are mainly minor and aim to improve clarity and biological context.

**Minor comments and clarifications**

1. **Background section: **Some morphological details described in the Background (e.g. pronotum characteristics and segment proportions) could not be fully found in the cited reference. We suggest adding one or two additional references to support these descriptions.
2. **Genome assembly description: **In the genome assembly section, it would be useful to clarify whether duplicate purging within *hifiasm* was applied, and at which level. In addition, explicitly stating that an unphased assembly pipeline was used would improve clarity, as referring only to the --primary option may not be clear to all readers.
3. **Nucleic acid extraction protocol: **The authors used the Automated MagAttract v2 protocol for HMW DNA extraction. A short explanation of why this protocol was chosen instead of the MagAttract protocol for small arthropods would be helpful.
4. **Typographical error: **On page 3, *Hempicrepidius hirtus* should be corrected to *Hemicrepidius hirtus*.

**Biological context: **Although we recognise the concise nature of genome notes, adding a small amount of biological interpretation would help readers better understand the results:

- The relatively high heterozygosity inferred from the k-mer analysis and GenomeScope plot (~0.96%) could be briefly mentioned.
- The assembly consists of 10 chromosomes  (9 autosomes and the X chromosome). A short comment on whether this chromosome number matches expectations for this species, genus, or family would be useful.
- The GC content of the genome is relatively low (~34–36%). Indicating whether this is typical for *Elateridae *or related beetle groups would add context.

In conclusion, this is a strong and well-executed genome note. Addressing the minor points above would further improve clarity and usefulness.

Are sufficient details of methods and materials provided to allow replication by others?

Yes

Is the rationale for creating the dataset(s) clearly described?

Yes

Are the datasets clearly presented in a useable and accessible format?

Yes

Are the protocols appropriate and is the work technically sound?

Yes

Reviewer Expertise:

Evolutionary Biology, Population Genomics and Genome Assembly

We confirm that we have read this submission and believe that we have an appropriate level of expertise to confirm that it is of an acceptable scientific standard.
